# The effects of a web-based decision aid on the intention to diagnostic self-testing for cholesterol and diabetes: a randomized controlled trial

**DOI:** 10.1186/1471-2458-14-921

**Published:** 2014-09-06

**Authors:** Gaby Ronda, Janaica EJ Grispen, Martine HP Ickenroth, Geert-Jan Dinant, Nanne K De Vries, Trudy Van der Weijden

**Affiliations:** Department of General Practice, Faculty of Health, Medicine, and Life Sciences, Maastricht University, P.O. Box 616, 6200 MD Maastricht, The Netherlands; Department of Health Promotion, Faculty of Health, Medicine, and Life Sciences, Maastricht University, Maastricht, The Netherlands; CAPHRI School for Public Health and Primary Care, PO Box 616, 6200 MD Maastricht, The Netherlands

## Abstract

**Background:**

Diagnostic self-tests are becoming increasingly available. Since the pros and cons of self-testing are unclear and neutral information on self-testing is lacking, two decision aids (DAs) on self-testing for cholesterol and diabetes were developed to support consumers in making an informed choice that is in line with their personal values. We aimed to evaluate the effect of the DAs on the intention to self-test for cholesterol or diabetes, as well as socio-cognitive determinants of that intention.

**Methods:**

1137 people of an internet panel with an intention to use a diagnostic self-test for cholesterol or diabetes were enrolled in a web-based randomized controlled trial consisting of four groups: a cholesterol intervention and control group and a diabetes intervention and control group. The study was conducted in September and October 2011. The intervention groups received an interactive online DA with general information on self-testing and test-specific information on cholesterol or diabetes self-testing, whereas the control groups received a limited information sheet with general information on self-testing. The intention to use a self-test for cholesterol or diabetes and perceived susceptibility, perceived severity, cues to action, perceived benefits, perceived barriers, self-efficacy and ambivalence towards self-testing were assessed directly after being exposed to the intervention or control information.

**Results:**

Follow-up measurement was completed by 922 people. Analyses showed a significant group by intention at baseline interaction effect within the diabetes condition. Further exploration of this interaction showed that a main group-effect was only observed among maybe-intenders; intention of participants in the intervention group did not change between baseline and follow-up, while intention slightly increased in the control group. We observed a significant main effect of group on cues to action in the cholesterol condition.

**Conclusions:**

We found limited effects of the DAs on intention and its determinants. Although the time spent on the DAs was limited, we might assume that our DAs contain neutral information on self-testing for cholesterol and diabetes. By implementing our DAs in real life among people who probably or definitely intend to use a self-test and by assessing weblog files, we might be able to determine the effectiveness of our DAs on self-test behaviour.

**Dutch trial register:**

NTR3149.

## Background

Diagnostic self-tests on body materials testing for conditions such as diabetes or elevated cholesterol levels are widely available to the general public [1–3]. Home self-tests can be bought in drugstores or via the Internet and can be used without the need to consult a health professional first. In home self-testing, the consumer is responsible for the execution of the test, the interpretation of the test result, and the follow-up behaviour based on this result. Diagnostic self-tests on body materials are often used; 18% of respondents participating in an online survey in the Netherlands had ever used a self-test in 2008. In total, 44% of these self-tests were considered to be home-tests whereas the remaining 56% were conducted as so-called ‘street corner’, ‘direct access’ or ‘home collect’ tests [4].

Self-diagnosis and therefore self-testing can be seen as a tool to stimulate autonomy and self-management as they can be used to take responsibility for one’s own health. However, since self-testing is a relatively new area in disease prevention and is still in a state of flux, the consequences of self-testing for individuals and society are currently unclear. Self-testing can be seen as a positive development i.e. self-testing may increase testing-rates resulting in more timely diagnosis and treatment, and self-testing can be used in the privacy of one’s home and is therefore convenient. However, self-testing might have negative consequences as well, as self-tests may be unreliable and relatively expensive. A false-negative result may delay treatment whereas a false positive result may lead to additional and more expensive medical tests, and testing without counselling may result in adverse medical or psychological outcomes [3, 5–8].

People intending to use a self-test are confronted with considering the pros and cons of self-testing. In previous unpublished observations we found that individuals who intend to perform a cholesterol or diabetes self-test simultaneously perceived benefits as well as barriers towards using a self-test, i.e., they hold positive and negative beliefs towards self-testing at the same time. Feelings of ambivalence can result in an uncomfortable state that people might want to resolve by making a decision [9]. To support people in making the choice whether or not to use a self-test, we developed two web-based decision aids (DAs) on self-testing for cholesterol and diabetes. DAs are aimed at supporting patients in making a screening or treatment choice that is the best choice considering their specific situation and matches their personal values and preferences. DAs have shown to improve patients’ knowledge on options for screening and diagnostic or therapeutic interventions, and to reduce decisional conflict [10]. Our DAs provide neutral information on diagnostic self-testing in general and on self-testing for cholesterol or diabetes specifically. The DAs were based on clinical practice guidelines, previous research on self-testing [2, 4, 11–14], the International Patient Decision Aid Standard (IPDAS) [15, 16], and the Health Belief Model (HBM) [17, 18].

The HBM was originally designed to explain relatively simply health behaviours, such as screening, which may be considered similar to self-testing. The HBM states that people will be inclined to engage in a health-related behaviour if they evaluate their susceptibility to and the severity of a particular condition of illness as high, and if they believe that a certain action can reduce this threat. Furthermore, they have to perceive more benefits than barriers regarding that behaviour. The presence of certain cues, for example the offering of free tests, can stimulate action. Additionally, they need to be confident about their ability to successfully perform the behaviour. Background variables such as socio-demographic characteristics and knowledge can influence the central concepts of the HBM and, as a result, health behaviour [17–20]. The aforementioned HBM concepts were included in our DAs, for instance information on the pros and cons of self-testing (perceived benefits and barriers). However, our DAs were not aimed at changing or influencing self-test behaviour and the concepts were not translated into behaviour change strategies.

The aim of this study was to evaluate the effect of the decision aids on the intention to self-test for cholesterol or diabetes as well as factors related to that intention by means of a randomized controlled trial (RCT) [21]. We addressed the following research questions: (1) ‘What is the effect on the intention to use a self-test for cholesterol or diabetes?, (2) ‘What is the effect on HBM-concepts of self-testing for cholesterol or diabetes, and (3) ‘Has there been a reduction in ambivalence towards self-testing for cholesterol or diabetes in the intervention conditions compared to the control conditions?’.

## Methods

### Ethical approval

The study was reviewed by the Medical Ethical Committee of Maastricht University Medical Centre which indicated that formal approval was not deemed necessary.

### Study design

A web-based randomized controlled trial was conducted in which the online DAs on cholesterol and diabetes were compared to short, non-interactive and non-test-specific information on self-testing. This resulted in four groups: one intervention and one control group consisting of participants with an intention to use a cholesterol self-test and one intervention and one control group with participants intending to use a diabetes self-test.

### Participants and procedure

Six thousand panelists were randomly selected from an Internet panel consisting of approximately 14,000 Dutch speaking members aged 12 years or older who have an e-mail address. This Internet panel is managed by Flycatcher [22], a Dutch institute for online research liaised with Maastricht University which was in charge of the enrollment of participants, the randomization, the assignment of the interventions, and the distribution of the questionnaires.

Panelists received an e-mail containing an invitation to participate in the study, providing a short description of the aim of the study, a link to the baseline questionnaire, and an expiration date. After one week, a reminder was sent to panelists who had not yet responded to the questionnaire.

**Figure 1:**
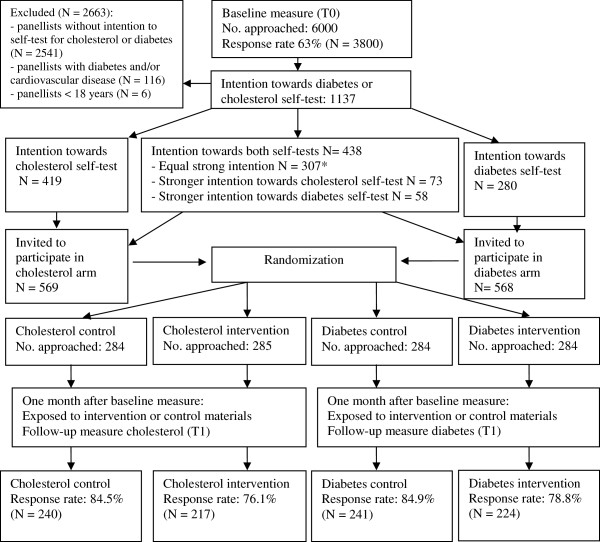
**Flowchart of RCT.** *To create groups of equal size in both study arms, 76 of the respondents with an equal strong intention were assigned to the cholesterol arm and 231 to the diabetes arm.

The researchers assigned the respondents of the baseline measurement with an intention towards diagnostic self-testing on cholesterol or diabetes to either the cholesterol or the diabetes study arm. If participants had an intention to do both, they were assigned to the test towards which they had the strongest intention (see Figure 1 for more details).

Subsequently, respondents in the cholesterol arm and respondents in the diabetes arm were randomly assigned to the intervention or control condition by the Flycatcher institute. Randomization of both groups into two groups of equal size was computer-generated with the random number generators function within the SPSS statistical package. Researchers and participants were blinded for randomization and participants did not know which intervention they received.

One month after filling out the baseline questionnaire, participants received an e-mail inviting them to participate in the follow-up questionnaire, tailored to either the cholesterol or the diabetes self-test. This e-mail contained a link to either the online DAs or the control information and informed participants to open this link, read the information, and after closing the link they were automatically routed to the follow-up questionnaire. After one week, a reminder was sent to panelists who had not filled out the questionnaire. Panelists received an incentive after completing the questionnaires, according to the norms held by Flycatcher. Socio-demographic variables such as age, gender, and level of education of each respondent were provided by Flycatcher.

A more detailed description of the methods is published elsewhere [21].

### Inclusion and exclusion criteria

Panelists were invited to participate in the RCT if they had an intention to maybe, probably or definitely use a cholesterol and/or diabetes self-test. People who reported having diabetes and/or cardiovascular disease (CVD), or who were younger than 18 years of age, were excluded from participation.

### Sample size

The sample size calculation described in this study was based on knowledge, although this is not an outcome measure of the current study. A related study will report on the results of outcomes on knowledge and associated factors. The sample size aimed for was 700 participants: 175 participants in each of the four groups [23]. This number was based on a Cochrane review on the efficacy of DAs in which an average absolute increase of 15% in knowledge scores was found [24]. Our sample size would allow a detection of a 15% absolute difference in knowledge with a power of 80% and an alpha of .05 (two-sided). Assuming a completion rate of 60% on the baseline questionnaire and 80% on the follow-up questionnaire [4, 12], an intention towards doing a cholesterol and/or a diabetes self-test of 42% [4], and considering an unknown number of participants to be excluded due to having CVD or diabetes, 6000 panellists were invited to participate in the baseline questionnaire [21].

### Intervention and control

The DAs on self-testing consisted of six core components.

The *homepage* provided information on the contents of the website and indicated that the DA is on diagnostic self-testing and therefore not suitable for individuals who already have diabetes or cardiovascular diseases.

The g*eneral information* section provided information about the different types of self-tests available.

*Information on cholesterol and diabetes self-testing* was specifically aimed at either cholesterol or diabetes and provided information about associated risk factors, a risk calculator, the types of self-tests available, information on reliability of self-tests, on the interpretation of the test-result, and follow-up actions.

A *value clarification tool* was included to support people in eliciting their personal values on self-testing by indicating whether they agreed or disagreed with twelve propositions representing advantages and disadvantages of diagnostic self-testing. People had to indicate which propositions were most important to them, after which a weighing scale indicated whether they were seemingly more inclined to self-test or not. Additionally, a FAQ-section (Frequently Asked Questions), a sitemap, a disclaimer, and contact information were provided.

The control condition consisted of a PDF file of one page which gave general information about self-testing (definition of a self-test and the types of self-tests that are available). Test-specific information or interactive elements were not included.

More information about the intervention and control condition is published in our study protocol [21].

### Measures

In this study, two consecutive questionnaires were used. In September 2011, the baseline questionnaire was sent to a random sample of the Flycatcher panel and was used for selecting consumers with an intention to maybe, probably or definitely use a diagnostic self-test for diabetes or cholesterol. The questionnaire consisted of questions on personal characteristics such as medical history of cardiovascular disease and diabetes, and intention towards doing a cholesterol or a diabetes self-test. The complete questionnaire is available elsewhere [25].

In October 2011, after participants had been assigned to the intervention or control condition and had been exposed to this condition, participants were invited to fill in the follow-up questionnaire. Participants with an intention to do a cholesterol self-test received questions on cholesterol self-testing, participants with an intention to do a diabetes self-test received a similar questionnaire, but addressing the diabetes self-test. The second questionnaire assessed the HBM-concepts and ambivalence. Our measurements of the HBM concepts were derived from validated questionnaires on other health behaviours [17–20]. We used the questionnaires in previous research on self-testing and found that perceived susceptibility, cues to action, perceived benefits, perceived benefits and self-efficacy were significant predictors of self-test use or the intention to use a self-test. They also contributed to the explanation of the variance of self-test use or the variance in the intention to use a self-test [12, 26]. In the current study, we framed the questions towards the specific disease and self-test under consideration. The complete questionnaires are available elsewhere [25].

### Outcomes

*Intention.* Intention to do a self-test in general and the intention to do a self-test for cholesterol and diabetes were measured (Table 1).

**Table 1:** **Outcome measures and timing of data collection**

Construct	Measures	No. of items	Item ^a^	Questionnaire ^b^
		(Cronbach’s α)	Answering options	
Intention	Intention towards self-testing	1	­Do you intend to use a cholesterol self-test in the future?	1, 2
			1 = Definitely not; 2 = Probably not; 3 = Perhaps, 4 = Probably,	
			5 = Definitely	
HBM-concepts	Perceived susceptibility:	2 (α = .747)	According to you, what are the chances that you will develop a cardiovascular disease?	1, 2
	the individual’s belief of the risk of contracting a certain disease			
			­1 = Very high; 2 = High; 3 = Not high/not low; 4 = Low; 5 = Very low;	
			6 = I already have cardiovascular disease	
			Recoded into: 0 = I already have cardiovascular disease; 1 = very low; 2 = low; 3 = not high/not low; 4 = high; 5 = very high	
	Perceived severity:	1	How severe do you think cardiovascular diseases are?	2
	the individual’s belief of the seriousness of a certain disease			
			1 = Very severe; 2 = Severe; 3 = Neutral; 4 = Not severe; 5 = Not severe at all	
			Recoded into: 1 = Not severe at all; 2 = Not severe; 3 = Neutral; 4 = severe; 5 = very severe	
	Cues to action:	7 (α = .713)	To me, a reason to use a cholesterol self-test would be If I have a medical complaint	2
	bodily or environmental events that trigger action such as education, symptoms, media			
			1 = completely disagree; 2 = disagree; 3 = neutral; 4 = agree; 5 = completely agree	
	Perceived benefits:	9 (α = .847)	According to me, performing a cholesterol self-test is important	2
	the individual’s belief that a certain action will reduce susceptibility or decrease seriousness			
			1 = completely disagree; 2 = disagree; 3 = neutral; 4 = agree; 5 = completely agree	
	Perceived barriers:	5 (α = .778)	The costs of a cholesterol self-test are a barrier to me	2
	the individual’s belief about the negative aspects/costs of a certain action			
			1 = completely disagree; 2 = disagree; 3 = neutral; 4 = agree; 5 = completely agree	
	Self-efficacy:	2 (α = .712)	Performing a cholesterol self-test is difficult	2
	the individual’s confidence in one’s capability to successfully perform a certain action			
			1 = completely disagree; 2 = disagree; 3 = neutral; 4 = agree; 5 = completely agree	
Ambivalence	Ambivalence	3 (α = .853)	The following questions concern the way you feel about a cholesterol self-test	1, 2
			With regards to doing a cholesterol self-test I have…..	
			1 = very definite feelings – 7 = very mixed feelings	

^a^Examples are provided regarding the cholesterol test. For the diabetes test the word ‘cholesterol’ is replaced by ‘diabetes’.

^b^Questionnaire 1: baseline; Questionnaire 2: one-month follow-up, directly after seeing intervention or control condition.

*HBM-concepts.* Possible determinants of self-test use were derived from the Health Belief Model (HBM), which included perceived susceptibility and severity, cues to action, perceived benefits and barriers, and self-efficacy (see Table 1 for definitions of the concepts).

*Ambivalence.* Ambivalence was assessed by using three items as proposed by Priester and Petty (Table 1) [27].

### Statistical analysis

Analyses were conducted using SPSS 19.0. An alpha of .05 was used for statistical significance. Basic descriptive statistics were used to describe the respondents’ socio-demographic characteristics. All analyses described below were conducted separately for cholesterol and diabetes. Multiple logistic regression analyses were conducted to identify potential dropout bias (with attendance versus dropout as the dependent variable and gender, age, education, intention at baseline, and group as independent variables).

To answer research questions 1 and 2 ‘What is the effect of the DA on the intention to use a self-test for cholesterol or diabetes?’ and ‘What is the effect of the DA on the HBM-concepts of self-testing for diabetes and cholesterol?’, multiple linear regression analyses were performed, with intention at T1 or the HBM-concepts as outcome variable, and group (control = 0; intervention = 1), intention at baseline and socio-demographics as covariates. To test for possible interaction effects, we included group*age, group*gender, group*level of education, and group*intention at baseline.

To answer our third research question, ‘Has there been a reduction in ambivalence towards self-testing for cholesterol or diabetes in the intervention conditions compared to the control conditions?’, multiple linear regression analyses were performed with ambivalence at T1 as outcome variable, and group (control = 0; intervention = 1), ambivalence at baseline, and socio-demographics as covariates. To test for possible interaction effects, we included group*age, group*gender, group*level of education, and group*ambivalence at baseline.

All predictors were first included in the model, after which non-significant interaction terms were excluded, starting with the interaction that was least significant. If significant interactions were found, sub-group analyses were performed.

## Results

### Participants

The baseline questionnaire was completed by 3800 panelists (response rate 63%). Of these panelists, 1137 (mean age 44 years, ranging from 18 to 88) indicated that they maybe, probably, or definitely intend to use a self-test for cholesterol or diabetes. Based on this intention, panelists were assigned to either a cholesterol-group (N = 569) or a diabetes-group (N = 568). Within the cholesterol and the diabetes-groups, participants were randomly assigned to the control group or the intervention group (Figure 1).

Follow-up measurement was completed by 922 people (mean age 44.4 years, ranging from 8 to 88): cholesterol intervention group (n = 217), cholesterol control group (n = 240), diabetes intervention group (n = 224), and diabetes control group (n = 241) (Figure 1).

There were no significant differences between participants who dropped out and participants who responded to our follow-up questionnaire with respect to gender, age, education and intention at baseline.

There were no significant differences between the intervention and control groups on socio-demographic characteristics, in neither the cholesterol nor the diabetes groups (Table 2).

**Table 2:** **Socio-demographic characteristics of respondents**

Characteristics		Cholesterol			
		Overall	Control	Intervention	Test-value [df]
N		457	240	217	
Age	Mean	44,2	43.6	44.8	F = 0.92 [1] ns
	(SD)	(12,9)	(12.5)	(13.4)	
	[Range]	[21;88]	[21–74]	[21–88]	
Gender	Male% (N)	37.0% (169)	36.7% (88)	37.3% (81)	*χ* ^2^ = 0.02 [1] ns
	Female% (N)	63.0% (288)	63.3% (152)	62.7% (136)	
Level of education^a^	Low% (N)	17.1% (78)	15.4% (37)	18.9% (41)	*χ* ^2^ = 1.12 [2] ns
	Middle% (N)	39.2% (179)	39.2% (94)	39.2% (85)	
	High% (N)	43.7% (200)	45.4% (109)	41.9% (91)	
**Characteristics**		**Diabetes**			
		**Overall**	**Control**	**Intervention**	**Test-value [df]**
N		465	241	224	
Age	Mean	44.6	45.3	43.9	F = 1.48 [1] ns
	(SD)	(12.8)	(13.3)	(12.1)	
	[Range]	[18–84]	[18–84]	[19–74]	
Gender	Male% (N)	38.5% (179)	36.1% (87)	41.1% (92)	*χ* ^2^ = 1.21 [1] ns
	Female% (N)	61.5% (286)	63.9% (154)	58.9% (132)	
Level of education^a^	Low% (N)	23.0% (107)	25.7% (62)	20.1% (45)	*χ* ^2^ = 2.36 [2] ns
	Middle% (N)	39,4% (183)	39.0% (94)	39.7% (89)	
	High% (N)	37.6% (175)	35.3% (85)	40.2% (90)	

Note: ^a^Low = primary and secondary school, Intermediate = intermediate vocational education, High = higher vocational education and university.

### The effect of the DA on the intention to use a cholesterol or diabetes self-test

#### Cholesterol

Analyses revealed no significant effect of group on the intention to use a cholesterol test (Table 3).

**Table 3:** **The effect of the DA on the intention, HBM concepts, and ambivalence to use a self-test for cholesterol**

Outcome ^a^	Intention T1						
Intention	B	95% CI	P-value				
Group^b^	-0.04	-0.18;0.09	0.54				
Intention T0	0.40	0.29;0.51	**0.00**				
Gender^c^	-0.08	-0.22;0.07	0.28				
Age	-0.00	-0.01;0.00	0.44				
Level of education^d^							
Dummy Low vs High	-0.02	-0.22;0.18	0.86				
Dummy Intermediate vs High	-0.03	-0.18;0.12	0.74				
**Outcome** ^**a**^	**Severity**				**Susceptibility**		
**HBM Concepts**	**B**	**95% CI**	**P-value**		**B**	**95% CI**	**P-value**
Group^b^	-0.06	-0.16;0.04	0.23		-0.05	-0.16;0.06	0.40
Intention T0	0.05	-0.03;0.13	0.19		0.06	-0.03;0.15	0.20
Gender^c^	0.10	-0.01;0.20	0.07		0.02	-0.10;0.14	0.75
Age	0.00	-0.00;0.01	0.16		0.00	-0.00;0.01	0.52
Level of education^d^							
Dummy Low vs High	0.09	-0.06;0.23	0.22		0.11	-0.06;0.27	0.20
Dummy Intermediate vs High	0.13	0.03;0.24	**0.02**		-0.05	-0.17;0.08	0.48
**Outcome** ^**a**^	**Benefits**				**Barriers**		
	**B**	**95% CI**	**P-value**		**B**	**95% CI**	**P-value**
Group^b^	0.08	-0.01;0.17	0.08		0.02	-0.08;0.12	0.66
Intention T0	0.21	0.14;0.28	**0.00**		-0.12	-0.20;-0.04	**0.00**
Gender^c^	0.05	-0.05;0.14	0.33		-0.09	-0.19;0.02	0.10
Age	0.00	-0.00;0.00	0.86		0.00	-0.00;0.01	0.14
Level of education^d^							
Dummy Low vs High	-0.12	-0.25;0.01	0.07		0.29	0.15;0.44	**0.00**
Dummy Intermediate vs High	0.06	-0.04;0.16	0.20		0.15	0.04;0.26	**0.01**
**Outcome** ^**a**^	**Cues to action**				**Self-efficacy**		
	**B**	**95% CI**	**P-value**		**B**	**95% CI**	**P-value**
Group^b^	0.11	0.02;0.21	**0.02**		-0.04	-0.17;0.10	0.60
Intention T0	0.05	-0.03;0.13	0.19		0.15	0.04;0.27	**0.01**
Gender^c^	0.06	-0.04;0.17	0.22		0.06	-0.08;0.21	0.39
Age	-0.01	-0.01;-0.00	**0.00**		-0.01	-0.01;0.00	0.06
Level of education^d^							
Dummy Low vs High	-0.07	-0.21;0.08	0.37		-0.31	-0.51;-0.11	**0.00**
Dummy Intermediate vs High	-0.02	-0.12;0.09	0.76		-0.16	-0.31;-0.01	**0.04**
**Outcome** ^**a**^	**Ambivalence T1**						
**Ambivalence**	**B**	**95% CI**	**P-value**				
Group^b^	0.02	-0.16;0.21	0.80				
Intention T0	NA	NA	NA				
Ambivalence T0	0.51	0.42;0.60	**0.00**				
Gender^c^	-0.25	-0.44;-0.06	**0.01**				
Age	-0.01	-0.01;0.00	0.21				
Level of education^d^							
Dummy Low vs High	-0.02	-0.29;0.25	0.88				
Dummy Intermediate vs High	-0.01	-0.21;0.19	0.95				

^a^If none of the interaction effects were significant, only the results of the model without interactions were reported.

^b^0 = control group; 1 = intervention group.

^c^0 = male; 1 = female.

^d^Low = primary and secondary school, Intermediate = intermediate vocational education, High = higher vocational education and university.

We found a significant effect of intention at baseline on intention at T1 towards using a cholesterol self-test, indicating that participants who had a high level of intention at baseline have a higher level of intention at T1 as compared to participants with a lower level of intention at baseline (Table 3).

#### Diabetes

Analyses revealed a significant group by intention at baseline interaction effect (Table 4). Sub-group analyses showed a significant negative effect of group on intention at T1 among participants who indicated to *maybe* intending to use a self-test for diabetes at baseline (B = -0.26, *p* = 0.00), but not among participants who indicated to probably or definitely intending to use a self-test for diabetes at baseline. Participants who indicated to maybe intending to use a self-test for diabetes at baseline in the control group, had a higher intention at T1 towards using a self-test after reading the information compared to participants in the intervention group, who did not change from their original intention score of 3 (*M* = 3.24, *SD* = 0.64; *M* = 3.01, *SD* = 0.80).

**Table 4:** **The effect of the DA on the intention, HBM concepts, and ambivalence to use a self-test for diabetes**

Outcome ^a^	Intention T1					
Intention	B	95% CI	P-value			
Group^b^	-0.92	-1.64;-0.19	**0.01**			
Intention T0	0.40	0.27;0.54	**0.00**			
Gender^c^	0.04	-0.10;0.19	0.55			
Age	-0.00	-0.01;0.01	0.87			
Level of education^d^						
Dummy Low vs High	-0.21	-0.40;-0.03	**0.03**			
Dummy Intermediate vs High	-0.03	-0.18;0.13	0.73			
Group* intention T0	0.23	0.03;0.43	**0.03**			
Outcome^a^	**Susceptibility**			**Severity**		
HBM Concepts	**B**	**95% CI**	**P-value**	**B**	**95% CI**	**P-value**
Group^b^	-0.05	-0.07;0.17	0.41	0.01	-0.12;0.14	0.92
Intention T0	0.11	0.02;0.19	**0.02**	0.16	0.06;0.25	**0.00**
Gender^c^	0.05	-0.07;0.18	0.40	0.11	-0.03;0.25	0.11
Age	0.00	-0.00;0.01	0.34	-0.00	-0.01;0.00	0.12
Level of education^d^						
Dummy Low vs High	-0.02	-0.18;0.15	0.85	-0.03	-0.21;0.15	0.78
Dummy Intermediate vs High	-0.06	-0.19;0.08	0.39	-0.05	-0.20;0.10	0.51
Outcome^a^	**Benefits**			**Barriers**		
	**B**	**95% CI**	**P-value**	**B**	**95% CI**	**P-value**
Group^b^	0.06	-0.03;0.15	0.19	0.03	-0.09;0.14	0.64
Intention T0	0.18	0.12;0.25	**0.00**	-0.16	-0.24;-0.08	**0.00**
Gender^c^	0.07	-0.02;0.17	0.14	-0.03	-0.15;0.09	0.60
Age	0.00	-0.00;0.01	**0.04**	-0.01	-0.01;0.00	**0.03**
Level of education^d^						
Dummy Low vs High	-0.07	-0.19;0.06	0.29	0.44	0.28;0.59	**0.00**
Dummy Intermediate vs High	-0.01	-0.12;0.09	0.81	0.14	0.01;0.27	**0.03**
Outcome^a^	**Cues to action**			**Self-efficacy**		
	**B**	**95% CI**	**P-value**	**B**	**95% CI**	**P-value**
Group^b^	-0.01	-0.10;0.09	0.92	0.05	-0.10;0.19	0.52
Intention T0	0.12	0.05;0.19	**0.00**	0.19	0.08;0.29	**0.00**
Gender^c^	0.08	-0.02;0.17	0.10	0.03	-0.12;0.18	0.68
Age	-0.00	-0.01;0.00	0.12	0.00	-0.00;0.01	0.32
Level of education^d^						
Dummy Low vs High	-0.02	-0.14;0.11	0.80	-0.39	-0.59;-0.20	**0.00**
Dummy Intermediate vs High	0.04	-0.07;0.14	0.47	-0.09	-0.25;0.07	0.29
Outcome^a^	**Ambivalence T1**					
Ambivalence	**B**	**95% CI**	**P-value**			
Group^b^	-0.17	-0.35;0.01	0.06			
Intention T0	NA	NA	NA			
Ambivalence T0	0.59	0.51;0.67	**0.00**			
Gender^c^	0.01	-0.18;0.20	0.94			
Age	-0.00	-0.01;0.01	0.69			
Level of education^d^						
Dummy Low vs High	-0.25	-0.50;-0.01	**0.04**			
Dummy Intermediate vs High	0.09	-0.11;0.29	0.39			

^a^If none of the interaction effects were significant, only the results of the model without interactions were reported.

^b^0 = control group; 1 = intervention group.

^c^0 = male; 1 = female.

^d^Low = primary and secondary school, Intermediate = intermediate vocational education, High = higher vocational education and university.

Additionally, we found a significant effect of level of education on intention towards using a diabetes self-test, indicating that participants with a low level of education had a lower intention at T1 compared with participants who have a high level of education (Table 4).

### The effect of the DA on HBM-concepts of self-testing for cholesterol or diabetes

Since we were primarily interested in the effects of group on HBM concepts, only the effects found of group (control and intervention groups) on these concepts will be reported. Additional significant effects are depicted in Tables 3 and 4.

#### Cholesterol

A positive significant effect of group on cues to action was found, suggesting that the intervention group perceived more cues to action than the control group (Table 3).

#### Diabetes

Analyses revealed no significant effects of group on HBM concepts (Table 4).

### The effect of the DA on ambivalence towards self-testing for cholesterol or diabetes

#### Cholesterol

We found no significant effect of group on ambivalence at T1 (Table 3).

We found ambivalence at baseline to be a significant predictor of ambivalence at T1, suggesting that participants who had a high level of ambivalence at baseline had a high level of ambivalence at T1 as well. Additionally, we found a significant negative effect of gender on level of ambivalence, indicating that men are more ambivalent towards using a cholesterol self-test than women (Table 3).

#### Diabetes

We observed a marginally significant effect of group suggesting that participants in the intervention group were less ambivalent than participants in the control group (Table 4).

Additionally, a significant main effect of ambivalence at baseline on ambivalence at T1 was found, indicating that participants who had a high level of ambivalence at baseline had a high level of ambivalent at T1 as well. Finally, we found a negative significant effect of level of education on ambivalence at T1, indicating that participants with a lower level of education are less ambivalent as compared to participants with a higher level of education (Table 4).

### Time spent on the website

In the diabetes intervention group, the mean time spent on the website was 169 seconds (range 7 – 2989 seconds, SD 284). In the diabetes control group, the mean time spent on the PDF was 74 seconds (range 13 – 771, SD 81). The mean time spent on the website in the cholesterol intervention group was 194 seconds (range 12–2352 seconds, SD 315). In the cholesterol control group, the mean time spent on the PDF was 89 seconds (range 9 – 791, SD 98). Differences between the intervention and control groups were statistically significant (p < .000).

## Discussion

Diagnostic self-testing is a new area in disease prevention that is still in a state of flux. There is an ongoing scientific debate regarding the pros and cons of self-test and the effects of self-testing on, for example, psychological, societal, and medical aspects, are still unclear. By developing DAs on self-testing for cholesterol and diabetes, we have tried to provide neutral information on self-testing that supports consumers in making an informed choice that is in line with their personal values. The aim of the present study was to evaluate the effect of the DAs on the intention to use a self-test for cholesterol or diabetes, as well as their effects on HBM variables and ambivalence towards self-testing.

Analyses revealed limited effects of being exposed to the DA on intention, the HBM variables, and ambivalence. Analyses on intention only showed a significant group by intention at baseline interaction effect within the diabetes condition. Participants who had indicated to maybe intend to use a self-test for diabetes at baseline, had a higher intention at follow-up in the control group compared to participants in the intervention group. The intention of these participants in the intervention group did not change between baseline and follow-up, whereas there was a slight increase in the control group. We did not find an effect at follow-up among participants who indicated to probably or definitely intend to use a self-test for diabetes at baseline. This might be a mere measurement effect implying that asking intention questions related to a particular health behaviour could in itself result in changes in level of intention and subsequent behaviour [28]. This measurement effect may have been outweighed by the information provided in the intervention group concerning pros as well as cons related to self-testing and thereby be restricted to the control group. An explanation for only finding this result in the group of *maybe* intenders might be that there is more variance within this group as compared to the group of people who probably or definitely intend to use a self-test. This group of maybe-intenders might include people who never actually thought about using a self-test but who did not want to reject the possibility of ever using a self-test beforehand as well as of people who actually doubt whether or not to use a self-test. These maybe-intenders might be easier to influence by merely asking questions as compared to people who already made up their mind, e.g. people who probably or definitely intend to use a self-test.

Analyses on the HBM variables revealed a significant effect of group on cues to action within the cholesterol condition. This group-effect on cues to action within the cholesterol condition reflects that the intervention group perceived more cues to action than the control group. One of the main aspects of our DAs was a risk calculator that addressed several risk factors for CVD and diabetes and provided an indication on the users’ risk for CVD or diabetes based on the answers provided. This might have led to a greater awareness of the risk factors associated with CVD within the intervention group which might have resulted in perceiving more cues to action in the intervention group than the control group.

We did not find any additional intervention effects on HBM concepts. The goal of our DAs was to provide neutral information on self-testing for cholesterol or diabetes and to support consumers in making an informed choice that is in line with their values regarding whether or not to use a self-test. Our DAs were not aimed at changing or influencing self-test behaviour and, as a result, within our DAs, the HBM concepts were not specifically addressed and translated into behaviour change strategies. Although the time spent on the DAs was limited, it might be suggested that we succeeded in providing neutral information on self-testing for cholesterol and diabetes.

We only found a marginally significant group effect on ambivalence towards using a diabetes self-test suggesting that participants in the intervention group were less ambivalent than participants in the control group. One of the main goals of DAs is to reduce decisional conflict and, consequently reduce ambivalence towards self-test use [10]. Our results suggest that we might have not achieved this. However, the majority of our sample consisted of participants who indicated to *maybe* intend to use a self-test for cholesterol or diabetes. According to the Precaution Adoption Process Model (PAPM), these maybe-intenders are people that are deciding about acting, but are undecided yet [29]. In our sample, the group of maybe-intenders may have included people who never thought about using a self-test before but who still want to have the opportunity to use a self-test in the future, and were therefore not personally engaged by the subject. These persons may report feelings of ambivalence, but because of their disengagement in the issue, they may not have been motivated to solve these feelings of ambivalence [9]. Ambivalence is experienced as being particularly unpleasant when people are confronted with the necessity to make a choice [9]. However, deciding about using or not using a diagnostic self-test is not a life-threatening decision and does not create an urge to solve these feelings of ambivalence [9]. Furthermore, within our study, participants were not forced to make a choice on whether or not to use a self-test, we merely asked if their *intention* to use a self-test changed. This may also have resulted in the lack of an urge to solve these feelings of ambivalence. Therefore, we may not have found any effects of our DAs on ambivalence.

### Study limitations and strengths

The results of the current study have to be interpreted in light of some strengths and limitations. The generalizability of our study findings might be limited because we used a random sample of an existing Internet panel. Compared to the general population in the Netherlands, our respondents were more often female, and more highly educated respondents were over-represented while low education levels were under-represented [30].

In most studies on the effectiveness of DAs, DAs are compared with usual care [10]. Since there is no usual care available in self-testing, we developed our own control materials. These control materials consisted of very limited information about self-testing containing the definition of self-testing and the different types of self-testing. All distinctive features of our DAs, e.g. interactive elements such as risk calculators and value clarification tools as well as test-specific information, were not included in the control materials. By doing this, we aimed to create a fair contrast between the intervention and control conditions. However, although there were significant differences between the intervention and control conditions in the time they spent on the DA respectively on the PDF, actual exposure to the DA might have been too low.

### Practical implications and future research

By developing DAs on self-testing for cholesterol and diabetes, we aimed to support consumers in deciding whether or not to use a self-test and to make a decision that is in line with their personal values. To determine whether we succeeded in achieving this aim, data on informed choice needs to be thoroughly analyzed, and will be reported elsewhere.

By using the Internet as a medium to deliver our DAs, we had the opportunity to potentially reach a large population and to include interactive and tailored elements in our DAs. However, the time spent on the DAs in our study was limited, and research on Internet delivered behaviour change interventions shows that the actual reach of Internet delivered interventions fall short of expectations. Evidence from studies on the efficacy of these Internet delivered interventions suggests that the actual use of and exposure to the intervention content is low and these exposure rates might be even lower when the intervention is implemented in real life. Additionally, the time spent on assessing the contents of Internet delivered interventions is limited which results in insufficient exposure to the intervention content [31–33]. Real life implementation of our DAs combined with the assessment of time spent on website and registration of which contents of our DAs are visited, need to determine which parts of our DAs actually reach the users.

The majority of our participants were *maybe*-intenders which might have resulted in a group of people who were not that motivated to use the DAs on self-testing. Since the information provided in our DAs was largely aimed at using the test and interpreting its result, these *maybe*-intenders might not have been interested in these subjects since they have not yet decided whether or not to use a self-test. Future research should assess the effects of our DAs among people who have actually decided to use a self-test and who arrived at the point of actually buying one. By targeting people who probably or definitely intend to use a self-test, it can be assessed whether the contents of our DAs are applicable within this group of intenders and whether the DAs have an effect on actual self-test behaviour.

## Conclusions

We found limited effects of the DAs on intention, HBM factors, and ambivalence. Although the time spent on the DAs was limited, we might assume that our DAs contain neutral information on self-testing for cholesterol and diabetes. By implementing our DAs in real life in combination with assessing time spent on website, registering the contents visited in the DA, and assessing its effects among people who probably or definitely intend to use a self-test we may be able to determine whether or not our DAs are effective in supporting consumers in making an informed choice that is in line with their personal values.
